# The DNMT1-associated lincRNA DACOR1 reprograms genome-wide DNA methylation in colon cancer

**DOI:** 10.1186/s13148-018-0555-3

**Published:** 2018-10-22

**Authors:** Saigopal Somasundaram, Megan E Forrest, Helen Moinova, Allison Cohen, Vinay Varadan, Thomas LaFramboise, Sanford Markowitz, Ahmad M Khalil

**Affiliations:** 1 Department of Genetics and Genome Sciences, Case Comprehensive Cancer Center, Case Western Reserve University School of Medicine, Cleveland, OH 44106 USA; 2 Case Comprehensive Cancer Center, Case Western Reserve University School of Medicine, Cleveland, OH 44106 USA

**Keywords:** Colon cancer, Epigenetics, lincRNAs, Tumorigenesis

## Abstract

**Background:**

DNA methylation is a key epigenetic mark in mammalian organisms that plays key roles in chromatin organization and gene expression. Although DNA methylation in gene promoters is generally associated with gene repression, recent studies demonstrate that DNA methylation in gene bodies and intergenic regions of the genome may result in distinct modes of gene regulation. Furthermore, the molecular mechanisms underlying the establishment and maintenance of DNA methylation in human health and disease remain to be fully elucidated. We recently demonstrated that a subset of long non-coding RNAs (lncRNAs) associates with the major DNA methyltransferase DNMT1 in human colon cancer cells, and the dysregulation of such lncRNAs contribute to aberrant DNA methylation patterns.

**Results:**

In the current study, we assessed the impact of a key DNMT1-associated lncRNA, DACOR1, on genome-wide DNA methylation using reduced representation bisulfite sequencing (RRBS). Our findings demonstrated that induction of DACOR1 in colon cancer cells restores DNA methylation at thousands of CpG sites throughout the genome including promoters, gene bodies, and intergenic regions. Importantly, these sites overlap with regions of the genome that become hypomethylated in colon tumors. Furthermore, induction of DACOR1 results in repression of *FOS* and *JUN* and, consequently, reduced AP-1 transcription factor activity.

**Conclusion:**

Collectively, our results demonstrate a key role of lncRNAs in regulating DNA methylation in human cells, and the dysregulation of such lncRNAs could emerge as a key mechanism by which DNA methylation patterns become altered in human tumors.

**Electronic supplementary material:**

The online version of this article (10.1186/s13148-018-0555-3) contains supplementary material, which is available to authorized users.

## Background

The epigenetic code is comprised of specific patterns of histone and DNA modifications [1–3], which cooperate to control gene expression without changing the underlying DNA sequence, typically through recruitment of various protein complexes to alter chromatin accessibility [4]. These epigenetic modifications are dynamic and critical for tissue-specific gene expression during development and cellular differentiation. Furthermore, many studies have now documented changes in the epigenetics landscape in human diseases, resulting in altered gene expression and, consequently, phenotype [4, 5]. In particular, there is global dysregulation of the epigenetic landscape in cancer cells, as compared to matched normal cells [4, 6–9]. These massive epigenetic alterations are thought to be critical events in the initiation and progression of tumorigenesis, and act in cooperation with somatic gene mutations to mediate tumor progression. Despite these extensive epigenetic changes in many tumor types, the underlying molecular mechanisms driving epigenetic changes are still emerging, as they are not always simply driven by mutations in key epigenetic modifiers [4–6, 9, 10].

One of the first epigenetic changes that were reported in cancer is DNA hypomethylation, which was first observed during colon tumorigenesis [11]. DNA methylation of cytosine residues within cytosine-phosphate-guanine site (CpG) dinucleotides to yield 5-methylcytosine is widespread throughout the mammalian genome and is catalyzed by DNA methyltransferases in response to various signals [12, 13]. DNA methyltransferase 1 (DNMT1) is the primary DNA methyltransferase responsible for maintaining DNA methylation patterns on newly synthesized DNA during cell division. Also, DNMT1, along with DNMT3a and DNMT3b, catalyzes de novo DNA methylation throughout development and cellular differentiation [12]. Aberrant DNA hypomethylation is an early epigenetic alteration in colon tumorigenesis that plays a key role in the transition from normal colon epithelium to hyperplastic epithelium by largely unknown mechanisms. During the course of tumorigenesis, DNA also becomes hypermethylated at specific gene promoters, often tumor suppressors, leading in some cases to gene repression. The mechanisms by which genome-wide changes in DNA methylation occur in colon cancer and other cancer types remain poorly understood.

In a recent publication from our laboratory, we demonstrated that many long non-coding RNAs are associated with DNMT1, suggesting that long non-coding RNAs (lncRNAs) have important functions in regulating genome-wide DNA methylation [14]. We further demonstrated that one such lncRNA, DNMT1-associated colon cancer repressed lncRNA 1 (DACOR1), is a positive regulator of DNA methylation in cell culture models of the disease. DACOR1 becomes repressed in colon tumors, and its re-expression in colon cancer cell lines resulted in re-methylation of specific CpG sites in gene promoters as assessed by the Illumina Infinium Human Methylation 450k beadchip microarray platform. However, these arrays have several limitations, including bias toward CpG methylation in promoter regions and potential false signals due to probe cross-reactivity and polymorphic CpGs. To identify the full impact of DACOR1 on DNA methylation on a genome-wide scale, we utilized reduced representation bisulfite sequencing (RRBS) [15], which led to the identification of thousands of CpG sites that are affected by DACOR1 induction. To determine if the CpG sites regulated by DACOR1 are relevant to colon cancer, we compared these sites to differentially methylated regions (DMRs) in two independent cohorts of colon tumors vs. normal colon tissues, and identified substantial overlap. Integration of these data sets with gene expression data sets, and functional studies of DACOR1 indicate a potential role of the ATF3 pathway in colon cancer (Additional file 1 and Additional file 2). Thus, these findings contribute to our understanding of how changes in DNA methylation in colon cancer affect gene expression, which is critical toward revealing the role of epigenetic changes in tumorigenesis and cancer progression [4, 9].

## Results

### Massive changes in genome-wide DNA methylation in colon tumors

**Fig. 1:**
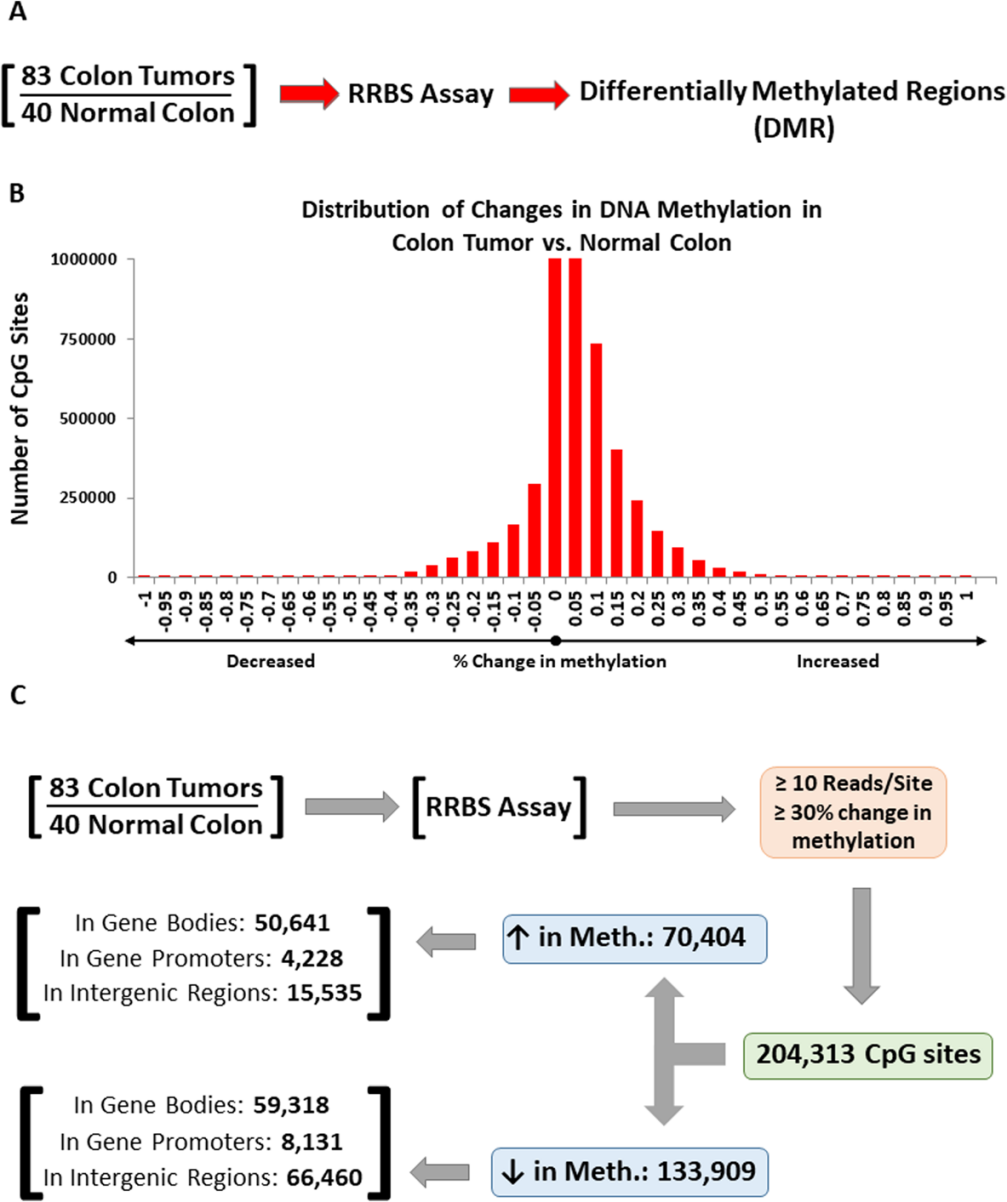
Genome-wide changes in DNA methylation in colon tumors. **a** Schematic of reduced representation bisulfite sequencing (RRBS) analysis workflow for 83 colon tumors vs. 40 normal colon samples. **b** Distribution of average change in DNA methylation in colon tumors vs. normal colon. **c** Distribution of differentially methylated CpG sites in colon tumors across the human genome. Using a stringent cutoff of > 30% change in DNA methylation, we identified over 204,000 CpG sites that are differentially methylated in colon tumors vs. normal colon. We further separated CpG sites that become hyper- vs. hypomethylated, and their genomic location (gene promoter, gene body, or intergenic region)

To identify specific regions of the genome that undergo changes in DNA methylation in colon tumors versus normal colon tissue, we performed DNA methylation analysis on 40 normal colon and 83 colorectal tumor samples using reduced representation bisulfite sequencing (RBBS) assay. Over six million CpG sites per sample were analyzed for differential DNA methylation, including CpG sites in promoters, gene bodies, and intergenic regions (Fig. 1a, b, Additional file 3: Dataset 1). Using a stringent cutoff of greater than 30% change in average methylation in tumors vs. normal samples, we identified 204,313 differentially methylated CpG sites (Fig. 1c, Additional file 3: Dataset 1). Of these 204,313 CpG sites, 70,404 (34.5%) showed increased methylation (hypermethylation), whereas 133,909 (65.5%) showed decreased methylation (hypomethylation) in tumors. To determine where in the genome these changes in DNA methylation occur, we aligned the differentially methylated CpG sites to hg19 RefSeq gene track from the UCSC genome browser to categorize CpG sites in promoter regions, gene bodies, and intergenic regions of the genome. Promoter regions were defined as areas in the genome starting 2 kb upstream of the transcriptional start site (TSS), gene bodies were defined as the genomic areas between the transcriptional start and end sites, and intergenic regions were all those regions that were not in the promoter or gene bodies. Using these criteria, we found extensive changes in DNA methylation not only in gene promoters but also in gene bodies and intergenic regions of the genome, often observing both increases and decreases in methylation within a single gene region. (Figure 1c, Additional file 3: Dataset 1).

**Fig. 2:**
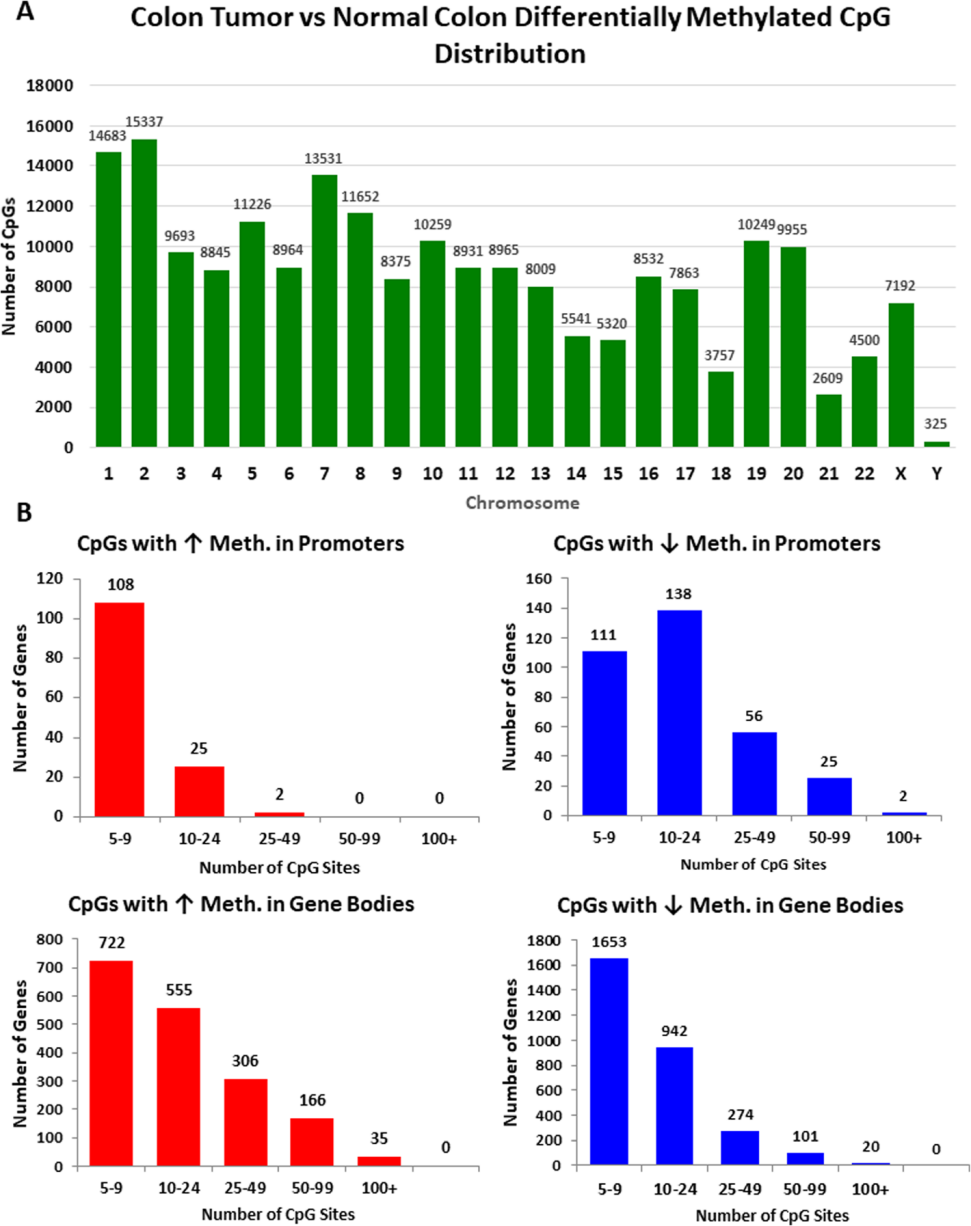
Changes in DNA methylation in colon tumors occur on all human chromosomes. **a** Number of differentially methylated CpG sites per human chromosome as assayed by RRBS assay. **b** Number of genes with either increased or decreased DNA methylation in promoters or gene bodies with number of CpG sites affected listed on the *x*-axis

Changes in CpG methylation in colon tumors versus normal colon (Fig. 1c, Additional file 4: Dataset 7) are observed on all chromosomes (Fig. 2a, Additional file 3: Dataset 1). We also determined the number of CpG sites that are affected in gene promoters and gene bodies in our cohort, and identified genes that show increased and/or decreased methylation in gene promoters and gene bodies (Fig. 2b, Additional file 3: Dataset 1). Remarkably, we found some genes that show substantial changes in DNA methylation at over 100 CpG sites in tumors vs. normal samples (Fig. 2b). Collectively, these data demonstrate extensive changes in DNA methylation in colon tumors in gene promoters, gene bodies, and intergenic regions of the genome. To further confirm these observations in an independent cohort, we performed similar analysis using public datasets from The Cancer Genome Atlas (TCGA), see below.

### DNA methylation is not a strong predictor of gene expression

**Fig. 3:**
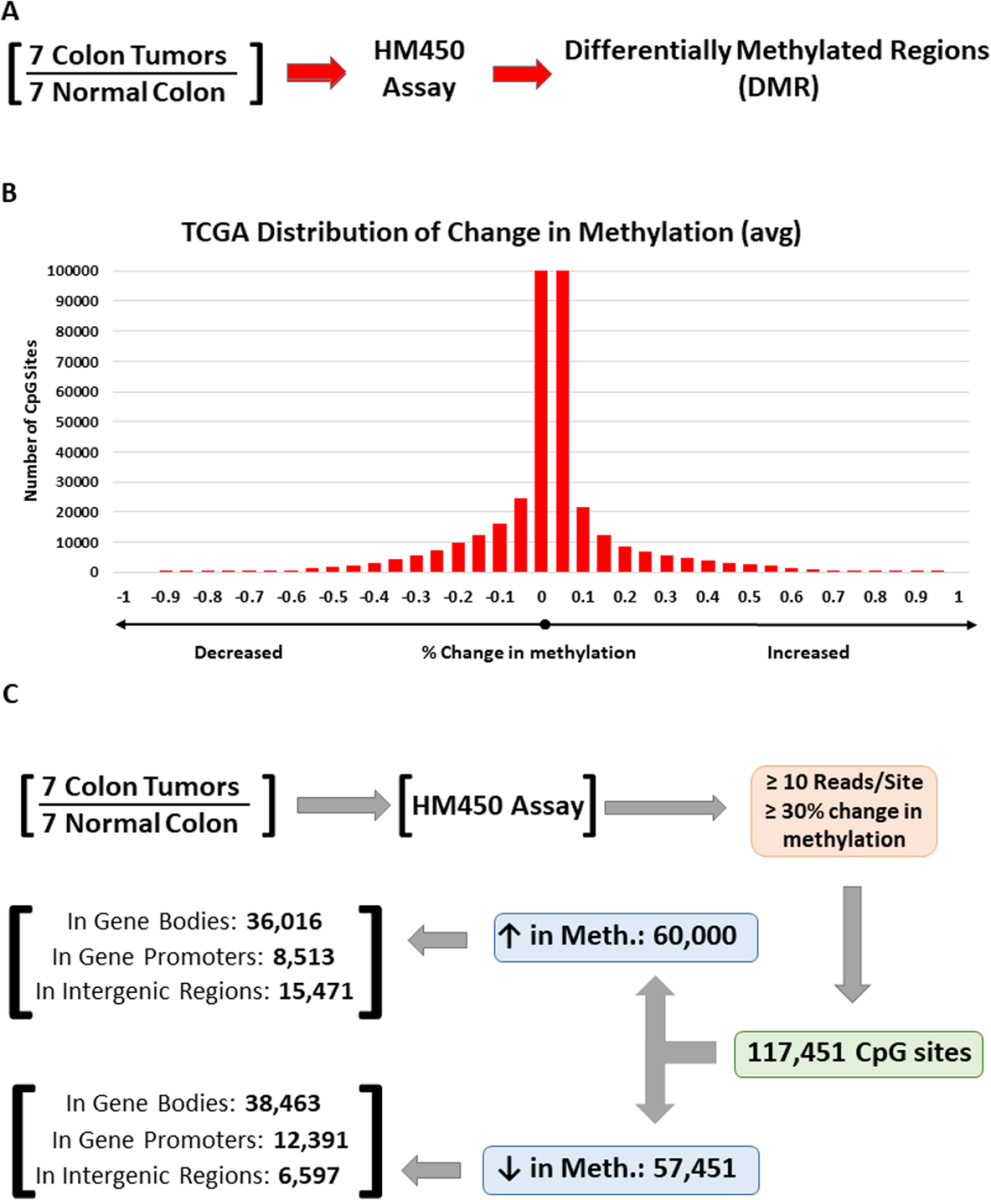
Confirmation of changes in DNA methylation in colon tumors using TCGA cohort. **a** Schematic of examining DNA methylation changes between seven colon tumors vs. seven matched normal tissues as assayed by HM450 methylation arrays. **b** Distribution of differentially methylated CpG sites between matched normal colon vs. colon tumors examined by HM450 methylation arrays. **c** Distribution of differentially methylated CpG sites in colon tumors across the human genome. Using a stringent cutoff of > 30% change in DNA methylation, we identified ~ 117,451 CpG sites that are differentially methylated in colon tumors vs. normal colon as assayed by HM450 arrays. We further separated CpG sites that become hyper- vs. hypomethylated, and their genomic location (gene promoter, gene body, or intergenic region)

**Fig. 4:**
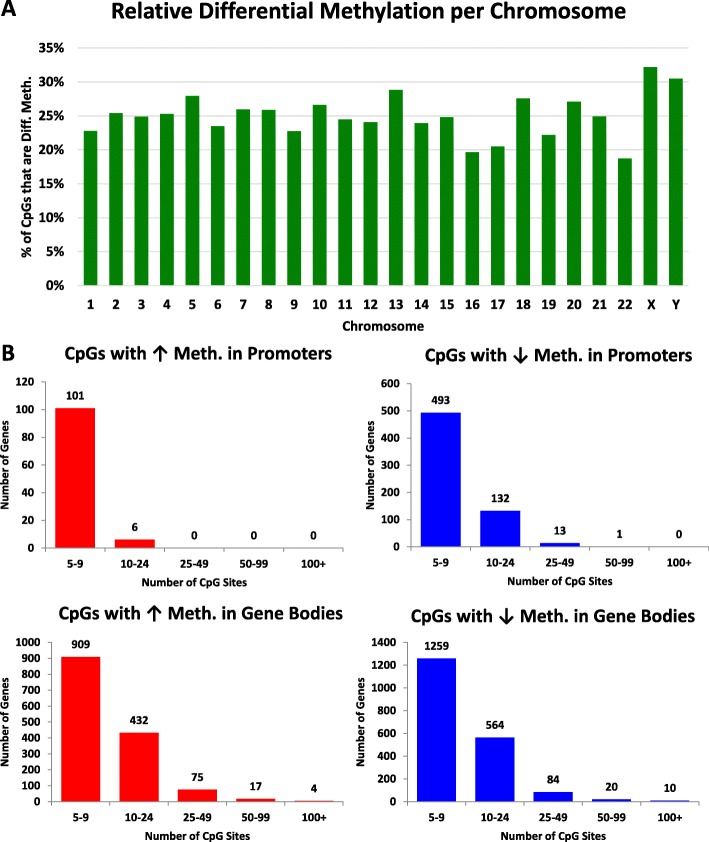
Differential methylation of CpG sites in gene bodies and promoter regions. **a** Frequency distribution of differentially methylated CpG sites in TCGA HM450 data is shown as a percentage of all CpG sites on each human chromosome. **b** Number of genes with differentially methylated CpG sites in either promoter or gene body regions at varying thresholds

To further test our observations in colon tumors vs. normal colon tissues, we downloaded DNA methylation data from TCGA from seven colon tumors that also had matched normal tissues. We restricted our analysis to tumor samples with matched normal tissues to account for differences that may result from human heterogeneity. TCGA DNA methylation studies were performed using HM450 arrays, which results in much less coverage than RRBS analysis. Nonetheless, these analyses supported our observations of both hypermethylation and hypomethylation occurring not only in promoters but also in gene bodies and intergenic regions (Fig. 3a–c, Additional file 5: Dataset 2). Also, we observed that the vast majority of changes in DNA methylation between tumors vs. normal samples show a difference of less than 50%, possibly due to tumor heterogeneity and/or aneuploidy (Fig. 3b, also see Fig. 1b). Furthermore, we did not observe a chromosomal bias of differentially methylated CpGs (Fig. 4a, Additional file 5: Dataset 2). Lastly, we identified genes with changes in either promoters or gene bodies that show hyper- and/or hypomethylation (Fig. 4b, Additional file 5: Dataset 2, Additional file 6: Dataset 8) to assess how these changes affect gene expression, see below.

**Fig. 5:**
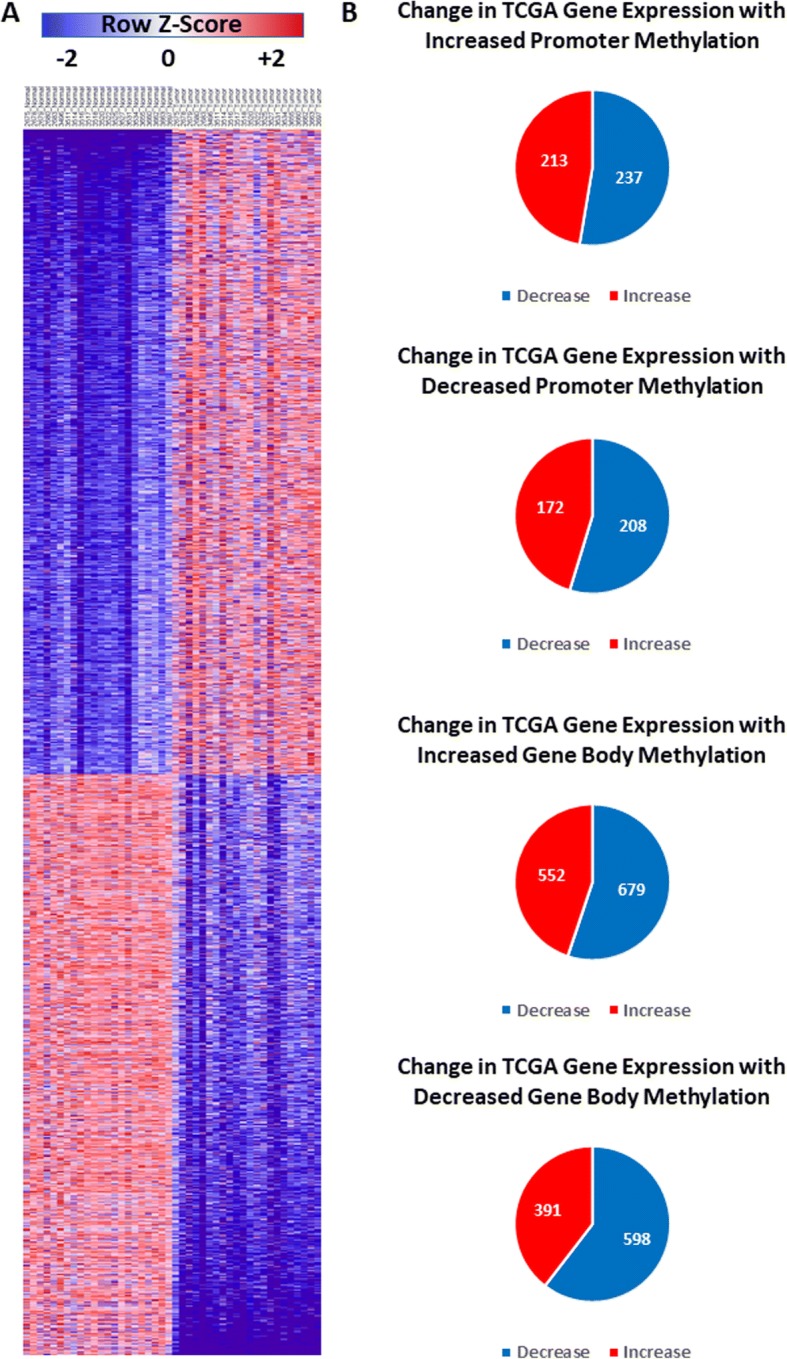
DNA methylation is not a strong predictor of gene expression. **a** Heatmap of differentially expressed mRNAs in colon tumors vs. normal colon using TCGA RNA-seq data. **b** Pie charts showing changes in gene expression following increases and/or decreases in methylation in differentially methylated CpG sites in gene promoters or gene bodies

Next, we wanted to determine the relationship between DNA methylation at specific genomic regions (promoters, gene bodies) and gene expression. To that end, we analyzed the expression of mRNAs in 22 colon tumors vs. 22 matched normal tissues (TCGA RNA-seq). We identified 2442 mRNAs that are differentially expressed (*p* < 0.05, ≥ 2-fold change) (Fig. 5a, Additional file 5: Dataset 2, Additional file 7: Dataset 5). We intersected genes with changes in DNA methylation in either promoter regions or gene bodies with their expression in tumors vs. normal colon and did not observe a strong correlation between DNA methylation and gene expression. For example, only ~ 52% of genes with increased DNA methylation in promoter regions showed decreased expression in tumors (Fig. 5b, Additional file 5: Dataset 2). These data demonstrate that DNA methylation is not sufficient to predict gene expression and that DNA methylation likely acts in conjunction with other changes in the epigenetic landscape to influence gene expression [9].

### Induction of DACOR1 results in genome-wide changes in DNA methylation

**Fig. 6:**
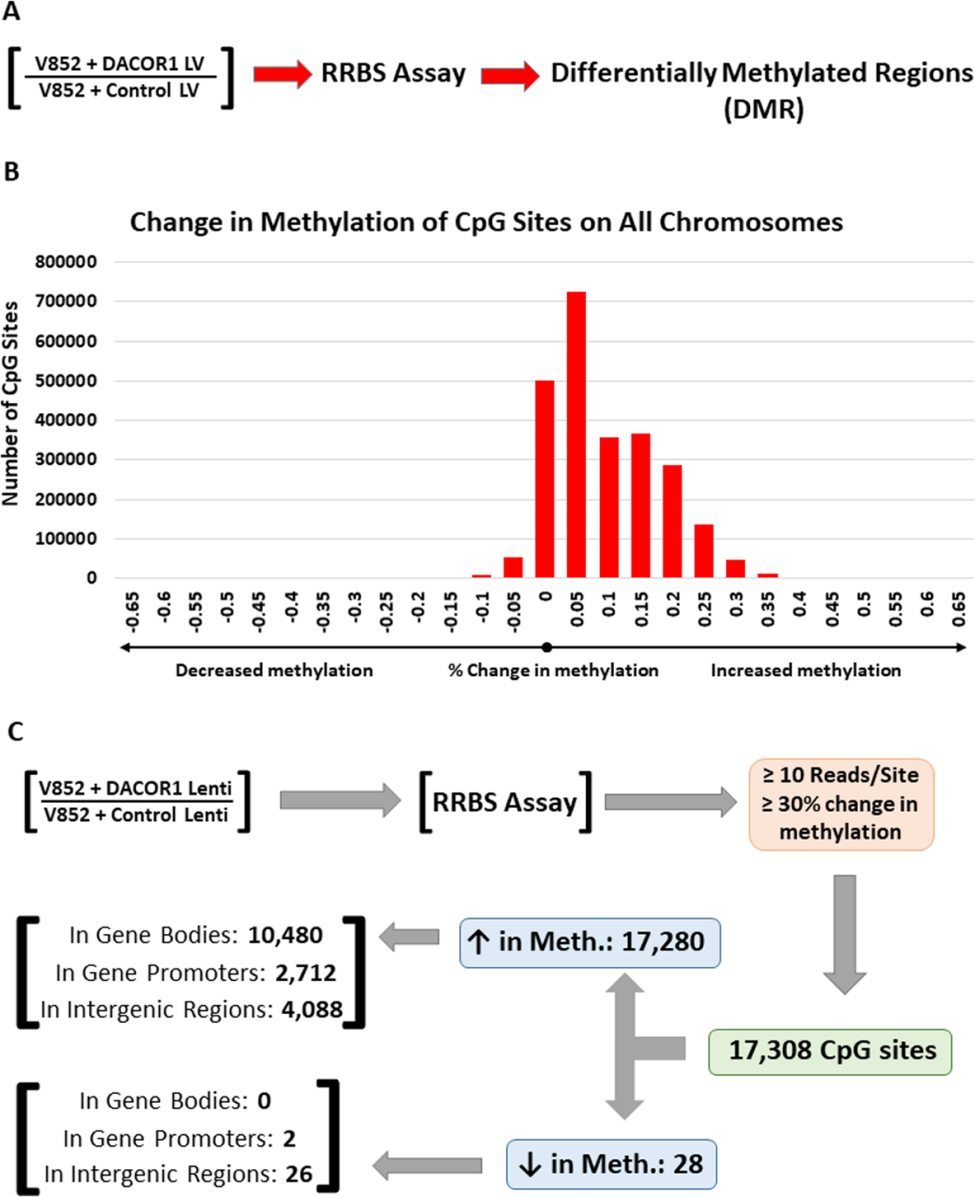
Re-expression of DACOR1 in V852 colon cancer cells results in genome-wide changes in DNA methylation. **a** The patient-derived colon cancer cell line V852 was transduced with either an empty vector lentivirus or a lentivirus containing the full-length DACOR1, and DNA methylation was examined by RRBS assay under both conditions (*n* = 3 of each condition). **b** Distribution of changes in DNA methylation in V852 cells in response to DACOR1 induction. **c** Over 17,000 CpG sites are affected by DACOR1 induction with the vast majority of sites (99.9%) showing gain of methylation

**Fig. 7:**
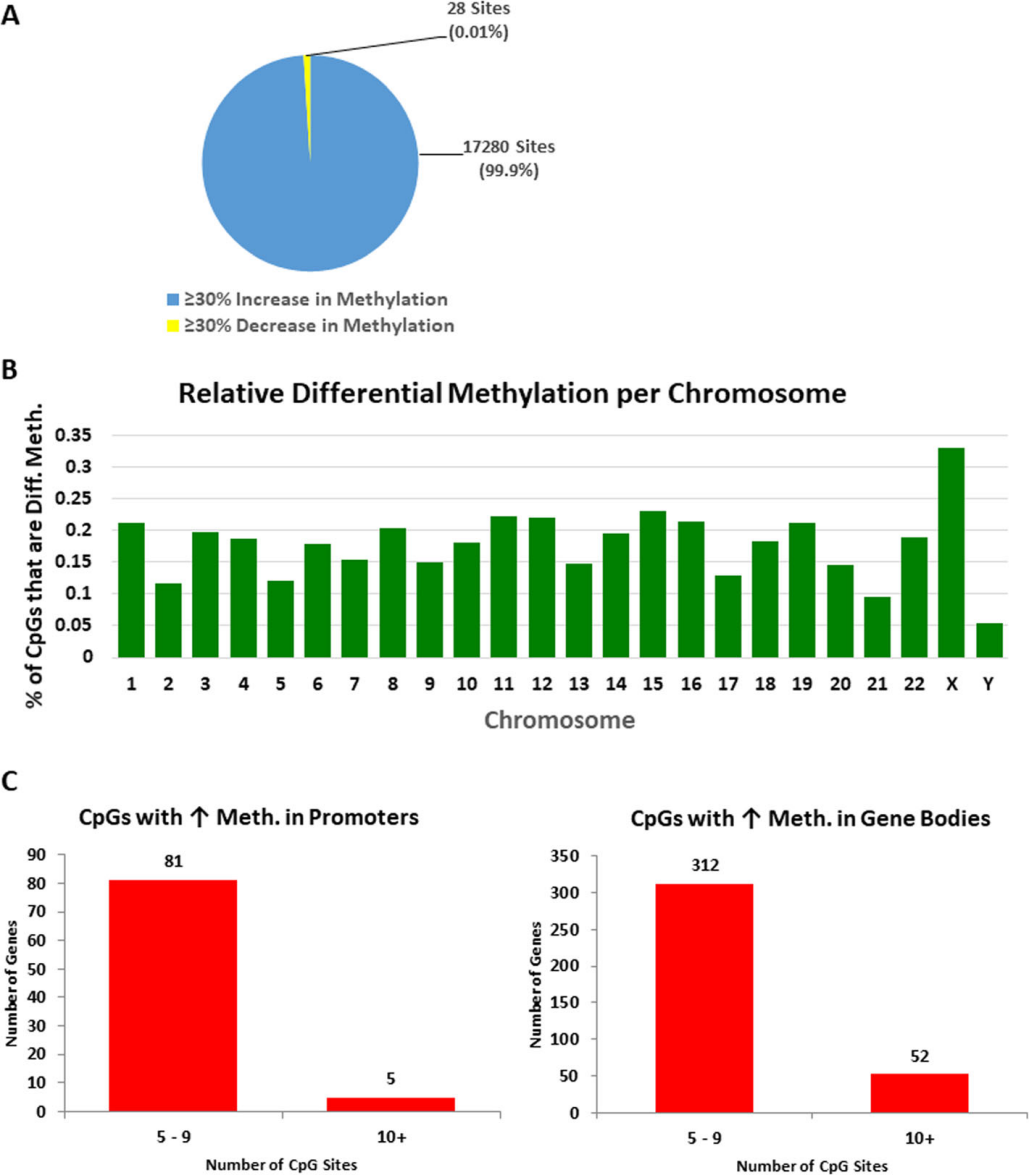
DACOR1 is a positive regulator of DNA methylation in colon cells. **a** Pie chart showing the number of CpG sites that gain or lose DNA methylation upon DACOR1 induction. **b** Distribution of differentially methylated CpGs across human chromosomes in response to DACOR1 induction. **c** Number of genes and number of differentially methylated CpGs in either promoters or gene bodies that are affected by DACOR1 induction.

We previously demonstrated that the expression of the DNMT1-associated lncRNA, DACOR1, results in increased CpG methylation as assessed by Illumina Infinium Human Methylation 450k beadchip microarray platform. These findings suggest a novel mechanism of RNA-directed DNA methylation in human cells and could potentially shed light on global hypomethylation observed in colon tumors. To further explore this mechanism, we first assessed the role of DACOR1 in regulating genome-wide DNA methylation by performing reduced representation bisulfite sequencing (RRBS) assay in a patient-derived colon tumor cell line (V852), which has low endogenous expression of DACOR1. We had previously produced V852 cells with stable re-expression of DACOR1 using a lentivirus containing the full cDNA sequence of DACOR1. By assessing genome-wide DNA methylation in this line, as compared to V852 cells transduced with a control lentivirus, we identified CpG sites that become differentially methylated in response to DACOR1 re-expression (Fig. 6a,  b, Additional file 8: Dataset 3). Strikingly, there was a strong bias toward increased DNA methylation upon DACOR1 induction as shown in Fig. 6b. Using a stringent cutoff of > 30% change in methylation between V852 control vs. DACOR1-expressing cells, we identified over 17,300 differentially methylated CpG sites. These differentially methylated CpG sites almost exclusively show an increase in DNA methylation; specifically, 17,280 (99.9%) of these CpG sites showed an increase in methylation in response to DACOR1 induction, while only 28 (0.01%) sites showed a decrease in methylation (Figs. 6c and 7a, Additional file 8: Dataset 3, Additional file 9: Dataset 6). These findings are in agreement with our previous findings that DACOR1 is a positive regulator DNA methylation in human cells.

To determine the genomic regions of DACOR1-mediated changes in DNA methylation, all affected CpG sites were mapped to individual human chromosomes; importantly, we did not observe any bias, with the exception of the Y chromosome showing only three affected CpG sites (Fig. 7b, Additional file 8: Dataset 3). We then aligned our list of differentially methylated CpG sites to the UCSC hg19 RefSeq gene track and identified specific genes that show increased DNA methylation in either gene promoters or gene bodies (Fig. 7c, Additional file 8: Dataset 3). By contrast, of the 28 CpG sites that showed a decrease in methylation in response to DACOR1 induction, two were mapped to promoter regions of two genes, while the remaining 26 CpG sites were within intergenic regions of the genome. Collectively, these findings demonstrate a key role for DACOR1 in positively mediating DNA methylation patterns genome-wide.

### DACOR1 regulates clusters of CpG sites in gene promoters

**Fig. 8:**
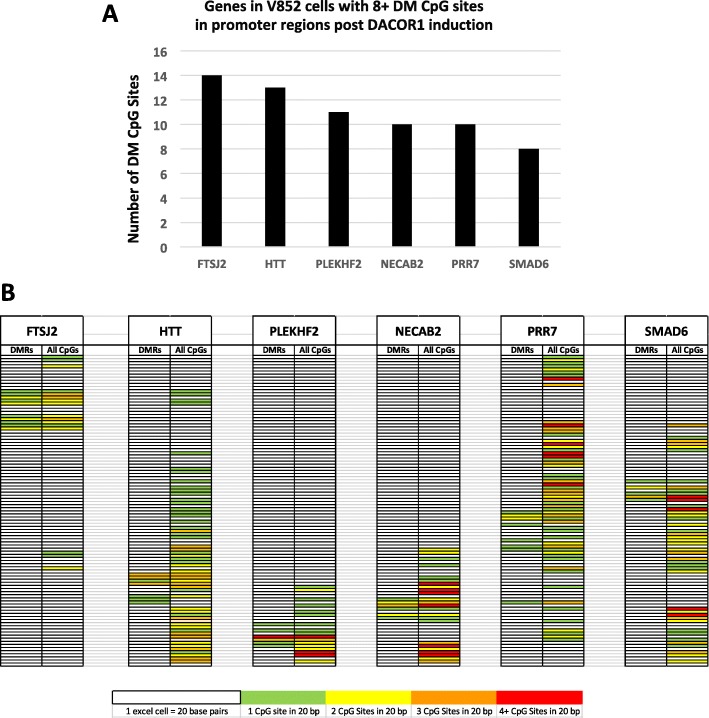
DACOR1-mediated DNA methylation affects clusters of CpGs in gene promoters. **a** Six genes with eight or more CpG sites in promoter region that undergo DNA methylation upon DACOR1 expression. **b** For each gene shown, we mapped the position of differentially methylated CpG sites compared to positions of all CpG sites within promoter regions

We identified 86 genes that gain DNA methylation in promoter regions upon DACOR1 induction in V852 cells (Fig. 7c, Additional file 8: Dataset 3). Six genes with eight or more CpG sites that gained DNA methylation upon DACOR1 induction in promoters were assessed for the distribution of these CpG sites relative to all CpG sites within promoter regions (Fig. 8a, Additional file 8: Dataset 3). Notably, for all six genes examined, the differentially methylated CpG sites clustered together in regions smaller than 200 base pairs. To test if DACOR1 regulates clusters of CpG sites within CpG islands, we mapped the position and density of affected CpG sites as well as all CpG sites for all six genes (Fig. 8b, Additional file 8: Dataset 3). Qualitatively, we observed that differentially methylated CpG sites are clustered together and are not dependent on the density or location of indexed CpG sites, suggesting that DACOR1 may target specific CpG clusters within gene promoters. To quantitatively assess if the clustering is not random, a MATLAB simulation (see “Methods” section) was performed to assess whether clustering, as measured by the number of adjacent differentially methylated CpG sites, was more than expected by chance. It was observed that for all 6 genes, such clustering in 100,000 simulations never surpassed what we observed in our RRBS data (*p* value 1 × 10^−5^).

### DACOR1-mediated DNA methylation in gene bodies is primarily in intronic regions

**Fig. 9:**
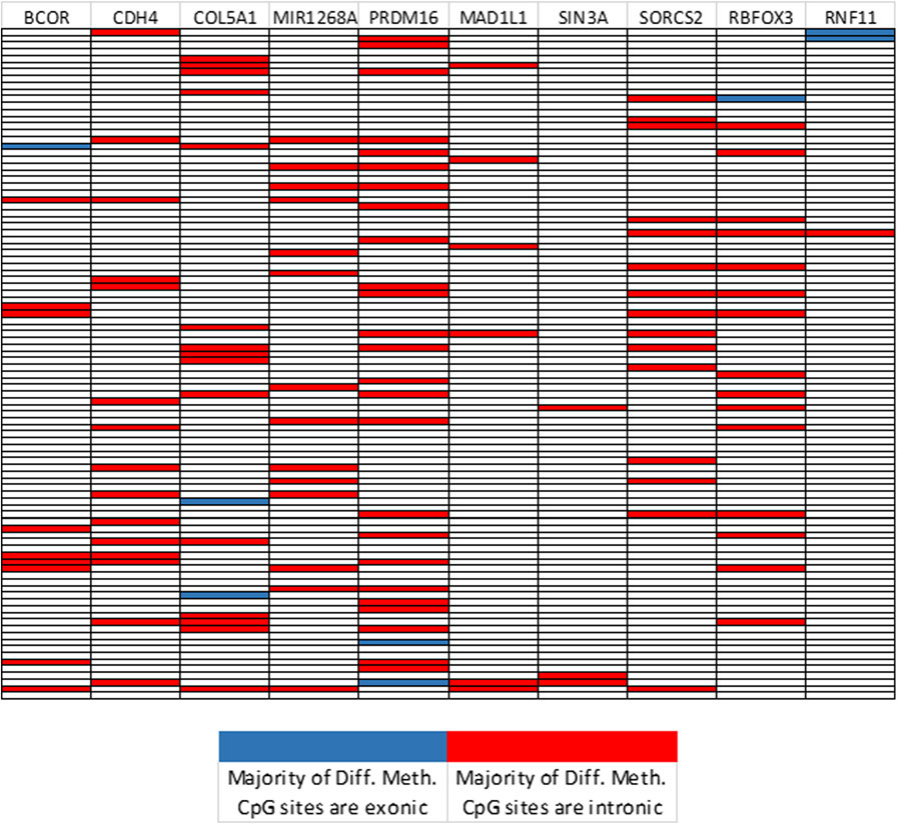
Changes in DNA methylation upon DACOR1 induction in gene bodies are primarily in introns. We examined the distribution of CpG sites in gene bodies that undergo changes in DNA methylation upon DACOR1 induction, and found that the majority of changes occur in intronic regions

Given the numerous CpG sites that become hypermethylated in response to DACOR1 expression within gene bodies, we wanted to determine if these CpG sites are predominantly exonic, intronic, or equally distributed. The top ten genes with the highest differential methylation rate in gene bodies were identified (Additional file 1: Figure S1A-B, Additional file 10: Dataset 4), and CpG sites in gene bodies of these top ten genes with most CpG sites were sorted into either intronic or exonic regions. We found that 93.23% of DACOR1-regulated CpG sites in gene bodies are within intronic regions of protein-coding genes (Fig. 9, Additional file 10: Dataset 4).

### DACOR1 regulates ATF pathway signaling in colon cancer

Given the extensive genome-wide changes in DNA methylation and the phenotypic effects of DACOR1 re-expression in colon cancer cells, we reasoned that DACOR1 expression is likely impacting key colon cancer-related pathways. First, we utilized The Broad Institute’s gene set enrichment analysis (GSEA) tool to identify critical pathways that are deregulated in colon cancer based on differentially expressed genes in colon tumors vs. normal colon identified in our analysis of RNA sequencing (RNA-seq) from TCGA (see Fig. 5a, Additional file 7: Dataset 5). Using this approach, several key pathways emerged including well-established colon cancer-related pathways, such as the Wnt/β-catenin pathway and p53-related signaling (Additional file 2: Figure S2). This indicated that our analysis successfully identified relevant pathways to colon tumorigenesis. We also identified enrichment of genes in the activated transcription factor 2 (ATF2) signaling pathway (*p* < 0.01, FDR = 0.057). This pathway further stood out as DACOR1 regulates key genes in this pathway both at the expression level (RNA-seq studies) as well as promoter and gene body methylation.Although the ATF signaling pathway is an important pathway in colon cancer, it remains relatively understudied.

The ATF family is a group of basic leucine zipper (bZIP) transcription factors, including ATF1, ATF2, ATF3, and other cyclic AMP response element-binding (CREB) family members, which are closely related to activating protein-1 (AP-1) complexes. Canonical AP-1 complexes are typically composed of Jun-Jun homodimers or Jun-Fos heterodimers; ATF proteins, particularly ATF3, can also bind to c-Jun to form alternative AP-1 complexes. The AP-1 transcriptional regulation program is known to play a role in multiple cellular processes in the context of cancer, including cell proliferation, differentiation, and regulation of both pro- and anti-apoptotic proteins. These complexes can either activate or repress transcription depending on the composition of the complex (e.g., Jun-Jun, Jun-Fos, Jun-ATF3), the promoter type, and the cell type, and can therefore act as either tumor suppressors or oncogenes in various cancer types.

**Fig. 10:**
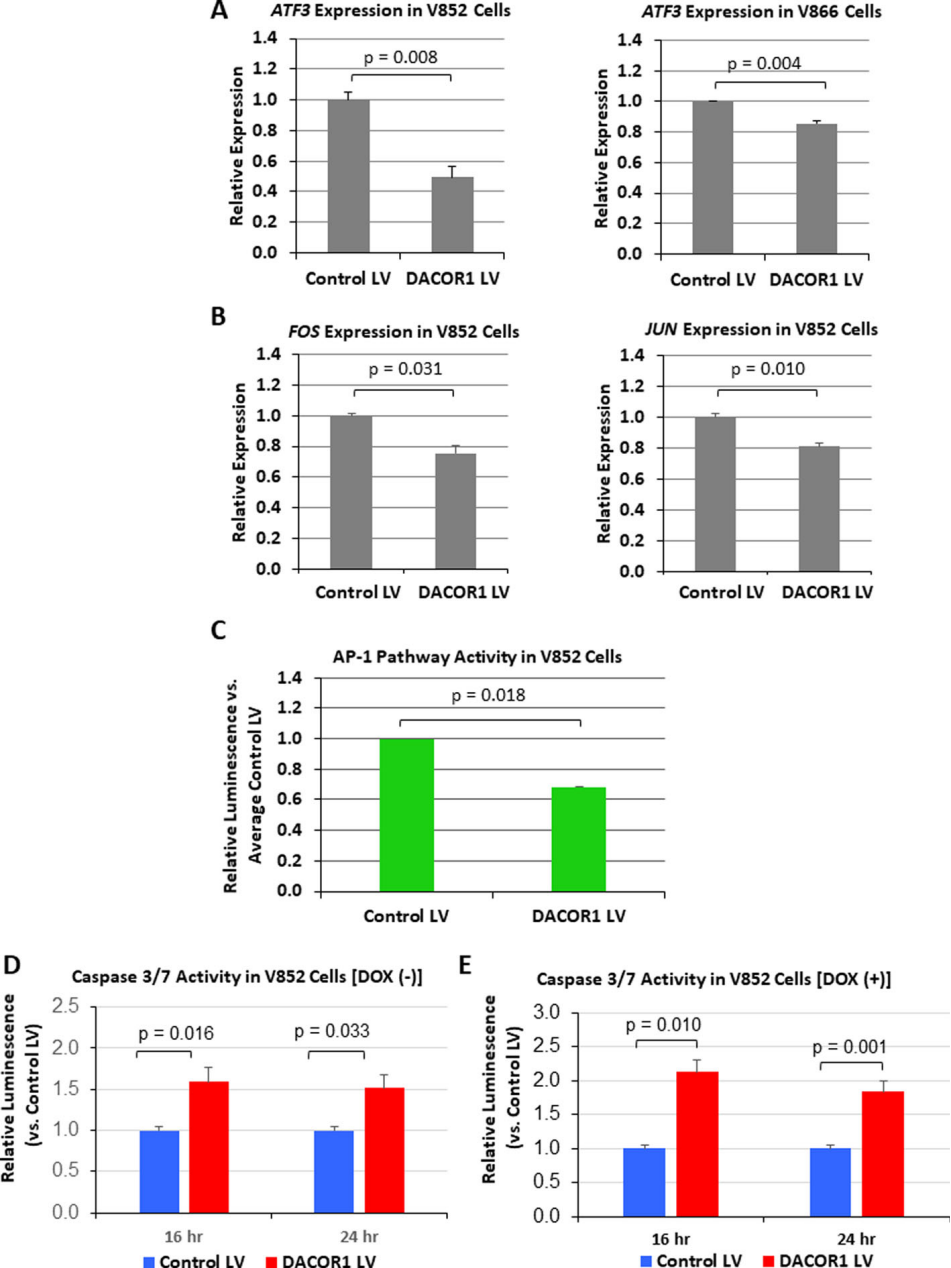
The AP-1 transcription factor network is altered by DACOR1 re-expression. **a**, **b** Quantitative RT-PCR of selected AP-1 pathway genes in V852 and V866 cells with either a control lentivirus or a lentivirus expressing the full-length DACOR1. DACOR1 expression results in the repression of ATF3, FOS, and JUN. **c** AP-1 activity is measured in V852 cells upon DACOR1 expression using a luciferase reporter. We found that the induction of DACOR1 results in attenuation of AP-1 activity, consistent with the changes in gene expression of ATF3, FOS, and JUN. **d**, **e** DACOR1 induction results in increased susceptibility to apoptosis

DACOR1 induction affects DNA methylation of ATF3 in both the promoter and gene body regions; thus, we decided to test the effect of DACOR1 induction on the expression of ATF3 and several key genes involved in ATF3 signaling pathway. First, we examined the effect of DACOR1 expression on ATF3 in two distinct patient-derived colon cancer cell lines, V852 and V866. In both cell lines, the induction of DACOR1 resulted in decreased expression of ATF3 (Fig. 10a). Next, we examined the effect of DACOR1 expression on FOS and JUN and observed significant decrease of both mRNAs upon DACOR1 induction (Fig. 10b). To test the functional consequences of the observed decrease in *ATF3*, *FOS*, and *JUN* gene expression, we performed an AP-1 luciferase reporter assay using a reporter plasmid containing tandem repeats of the AP-1 transcription factor consensus binding site coupled to firefly luciferase. AP-1 pathway activity was significantly decreased in DACOR1-expressing cells (Fig. 10c), consistent with the observed changes in gene expression. As discussed above, AP-1 transcription factor complexes are involved in the regulation of anti-apoptotic genes in the context of colon cancer. To test whether dysregulation of the AP-1 pathway activity in DACOR1-expressing cells results in changes in apoptosis, we performed a caspase 3/7 cleavage assay. We observed a significant increase in apoptosis in DACOR1-expressing cells relative to control cells (Fig. 10d); this effect was also observed upon treatment with doxorubicin (Fig. 10e), a known pro-apoptotic agent in colon cancer. Taken together, these results suggest a possible role for DACOR1 in the regulation of the ATF pathway and downstream AP-1 complex formation. Thus, repression of DACOR1 during colon tumorigenesis may lead to resistance to apoptosis, further promoting cancer progression.

## Discussion

In this study, we characterized differential DNA methylation patterns across colon tumors versus normal colon using RRBS technology, which led to the identification of over 204,000 differentially methylated CpGs in various regions of the genome (Additional file 11: Dataset 10). These data demonstrate extensive genome-wide changes in the epigenome during colon tumorigenesis; however, the underlying mechanisms of these global changes are largely unknown. Our previous and current data provide evidence for a lncRNA-mediated regulation of DNA methylation. Specifically, we provide evidence that a DNMT1-associated lncRNA, DACOR1, regulates DNA methylation at thousands of CpG sites across the genome. Our current model proposes that when DACOR1 becomes repressed during colon tumorigenesis, this loss of expression leads to loss of DNMT1 targeting and/or loading to specific regions of the genome. This model is supported by our experimental data where the re-expression of DACOR1 leads to re-methylation of more than 17,300 CpG sites. These data clearly suggest that DACOR1 is a positive regulator of DNA methylation in human colon cells. It is possible that other DNMT1-associated lncRNAs also regulate DNA methylation either positively or negatively. However, experimental evidence is needed to demonstrate the role of other DNMT1-associated lncRNAs in regulation of the epigenome.

The functional consequences of DNA methylation on gene expression are dependent upon the genomic location of DNA methylation (promoter, gene body, or intergenic) and cellular context. DNA methylation at CpG sites within promoters is frequently associated with gene silencing; however, recent studies suggest that there are additional consequences of promoter methylation. For example, DNA methylation in gene promoters has been shown to recruit activating transcription factors and chromatin-modifying complexes, demonstrating that the effects of DNA methylation on gene expression depend on additional epigenetic readers.

We also identified differentially methylated CpG sites that are outside of gene promoters, particularly within gene bodies. While some studies suggest that DNA methylation levels within gene bodies are positively correlated with gene expression, other studies suggest that gene body methylation exists as a mechanism for repression of spurious transcription contributing to transcriptional efficiency and genome stability. Thus, the consequences of aberrant gene body methylation, particularly in the context of cancer, remain to be elucidated. In addition, further studies are needed to determine the interplay between DNA methylation and other epigenetic marks to fully comprehend the functional consequences of changes in DNA methylation, both in normal cellular development and in disease states such as cancer.

A major challenge in the analysis of genome-wide DNA methylation in gene promoters and gene bodies in tumors is that hundreds of genes have both hyper- and hypomethylated CpGs; thus, it is challenging to classify these genes as hypo- or hyper-methylated without an arbitrary cutoff. This leads us to hypothesize that specific CpGs, based on their location in gene promoters or gene bodies, have stronger influence on gene expression than others. With the recent advent of genome editing technologies, it is now possible to perform experiments to test this hypothesis, and begin dissecting the effects of specific CpGs on gene expression.

## Conclusion

Although DNA methylation has been studied for several decades in both health and disease, it remains unclear how this epigenetic mark contributes to gene regulation. With the advent of genome editing technologies, we now have the molecular tools to precisely alter specific CpG sites alone or in combinations to determine how they contribute to gene regulation. In summary, we have documented genome-wide changes in DNA methylation patterns in colon cancer and provided evidence that lncRNAs play a role in mediating DNA methylation. These studies should pave the way for a better understanding of how epigenetic alterations occur during tumorigenesis, and their contribution to disease state.

## Methods

### Cell culture

Patient-derived colon cancer cell lines were maintained in MEM 2+ media (2% FBS, 2 mM glutamine, 1 μg/mL hydrocortisone, 10 μg/mL insulin, 0.86 ng/mL selenium, 2 μg/mL transferrin, 50 μg/mL gentamicin) at standard conditions (37 °C, 5% CO_2_) and passaged approximately every 3–5 days by trypsinization.

### Reduced representation bisulfite sequencing assay and data analysis

Bisulfite treatment of DNA converts unmethylated cytosine residues into uracil, which enable the identification of methylated vs. unmethylated CpGs. Reduced representation bisulfite sequencing (RRBS) is an alternative to whole genome bisulfite sequencing that allows analysis of DNA methylation on a genome-wide scale, offering significantly increased CpG coverage versus array-based methods.

To identify DACOR1-regulated CpG sites, genomic DNA (gDNA) was isolated from three biological replicates of V852 cells stably transfected with a control lentiviral vector or a lentivirus expressing the full-length DACOR1 using the QIAGEN DNeasy Blood and Tissue Kit. Then, 100 ng of gDNA from each sample was digested with MspI restriction enzyme (New England Biosciences) at 37 °C overnight. Library preparation (consisting of end-repair, 3′-adenylation, and adapter ligation) was carried out using the NEXTflex Bisulfite Sequencing kit protocol (BIOO Scientific) with 6 NEXTflex Bisulfite-Seq barcodes. Resultant libraries were manually size-selected using gel electrophoresis and gel extraction (reagents included in NEXTflex Bisulfite Sequencing kit) to obtain fragments in the 200–400-bp range. Resultant fragments were bisulfite treated and purified using the QIAGEN EpiTect Bisulfite kit protocol. Enrichment PCR was performed on bisulfite-treated sample using NEXTflex 0.5 μM primer mix, 0.4 mM dNTPs, 0.5 M betaine, and 0.2 μL Platinum Taq Polymerase with 1X PCR buffer and 2 mM MgCl_2_ (Invitrogen). Enriched libraries were purified using Agencourt AMPure XP beads (Beckman Coulter) and analyzed for purity and proper size selection using the Agilent High Sensitivity DNA bioanalyzer chip. Final libraries were sequenced at McGill University and Génome Québec Innovation Center (Montreal, QC, Canada) on the Illumina HiSeq 2000 using paired-end 100-bp reads. Resultant reads were mapped to the genome (hg19), and differentially methylated CpGs were identified. Specifically, CpG sites that showed fewer than ten reads in any of the V852 Control-LV or V852 DACOR1-LV samples were trimmed from the dataset, and methylation averages for Control LV and DACOR1 LV samples were calculated. CpG sites that showed a change in average methylation of at least 30% between the two samples were designated as differentially methylated (Additional file 9: Dataset 6).

Similar RRBS preparation and data analysis was performed on gDNA obtained from normal vs. colon tumor samples presented in Fig. 1. Colon tumor and normal colon samples that showed fewer than ten reads for a specific CpG site were trimmed from the dataset for that CpG site. An average colon tumor and normal colon methylation value was calculated for each CpG site. CpG sites that presented a change in average methylation of at least 30% between colon tumors vs. normal colon samples were designated as differentially methylated (Additional file 4: Dataset 7). Differentially methylated CpG sites from Illumina HM450 (TCGA Samples) between primary tumors and normal samples are in Additional file 6: Dataset 8. Demographic and phenotypic data for the cohort in Fig. 1 is presented in Additional file 12: Dataset 9. Affected CpG sites according to their location in CpG islands, shores or shelves are in Additional file 11: Dataset 10.

### RNA isolation and cDNA preparation

Trizol reagent (Life Technologies) was added directly to cell culture plate wells (1 mL per 1 × 10^7^ cells) and incubated at room temperature for 5 min. Phenol/chloroform RNA isolation proceeded using the QIAGEN RNeasy Mini Kit protocol with DNase digestion. RNA was quantitated using the NanoDrop 1000 Spectrophotometer. cDNA was prepared using the RNA to cDNA EcoDry™ Premix kit protocol (Clontech). cDNA was diluted to 2 ng/μL for quantitative real-time PCR (qRT-PCR) analysis.

### Quantitative real-time PCR

SYBR Green qRT-PCR was performed using 10 ng cDNA, 2× Maxima SYBR Green/ROX qPCR Master Mix (Thermo Scientific), and 0.3 μM forward primer and reverse primer. Samples were cycled using the standard SYBR Green protocol on an ABI StepOne qPCR instrument and analyzed using the comparative cycle threshold (*C*_T_) method to obtain relative expression quantities.

### Caspase 3/7 activity assay for apoptosis

Cells were plated in quadruplicate on two separate 96-well plates at a density of 5000–10,000 cells/well and allowed to incubate overnight (~ 16 h). Additional cells were plated in two sets of triplicates for DOX(+) and DOX(−) control to verify apoptosis induction. Then, 100 μM doxorubicin (DOX) stock solution (prepared from doxorubicin hydrochloride; Fisher Scientific, Cat #ICN15910101) was diluted to 1 μM in media and added to each test well and DOX(+) control wells; 100 μL fresh media was added to DOX (−) control wells. At 10 h and 24 h post DOX treatment, 100 μL of caspase 3/7 GLO reagent (Promega caspase 3/7 GLO Assay) was added to each test well, incubated at room temperature for 15 min, and read on the Wallac Victor2 1420 Multilabel Counter using the luminometer setting.

### AP-1 luciferase reporter assay

3xAP1pGL3 (3xAP-1 in pGL3-basic) was a gift from Alexander Dent (Addgene plasmid # 40342). The 3x AP-1 reporter construct contains three tandem AP-1 binding sites (TGACTCA) upstream of a minimal promoter fragment in the firefly luciferase reporter plasmid pGL3-basic backbone (36). pcDNA 3.1 Hygro Renilla Luciferase vector was a gift from William Schiemann (Case Western Reserve University). 3xAP-1 firefly luciferase reporter and renilla luciferase control plasmids were co-transfected in a 3:1 ratio in V852 Control LV and V852 DACOR1 LV cells using the Lipofectamine 3000 transfection protocol (Invitrogen). Firefly and renilla luciferase signal was read 36 h post-transfection using the Dual-Glo® Luciferase Assay System (Promega). Results were expressed as relative firefly:renilla signal ratios.

### Western blots

Protein was isolated from approximately one million cells using RIPA buffer and quantitated using the Pierce BCA Protein Assay kit protocol (Thermo Fisher Scientific). SDS-PAGE was performed using 4–20% Mini-PROTEAN® TGX™ Precast Protein Gels (BIO-RAD), and resultant proteins were transferred to nitrocellulose membrane (Thermo Fisher Scientific). Rabbit α-Histone H3 and Rabbit α-cleaved caspase 3 were obtained from Cell Signaling Technology, rabbit α-Phospo-c-Jun was obtained from Abgent, and goat α-rabbit-HRP conjugate secondary antibody was obtained from Abcam. Blots were blocked in appropriate blocking solution according to antibody product insert (5% milk in PBST for α-H3 and α-cleaved caspase 3; 3% BSA in PBST for α-Phospho-c-Jun). Following secondary antibody incubation, blots were developed using SuperSignal West Pico Chemiluminescent Substrate Solution and CL-X Exposure Film (both Thermo Fisher Scientific).

### Differentially expressed genes in tumors vs. normal colon analysis

RNA-sequencing (RNA-seq) raw data for 22 colon tumors and 22 adjacent normal tissues were obtained from publicly available TCGA database. Fragments per kilobase of transcript per million mapped reads (FPKM) values for each gene in each samples was calculated and differentially expressed genes were identified based on a greater than a twofold change and *p* < 0.05 in a two-tailed *T* test. This analysis led to the identification of 2443 differentially expressed genes between tumor and normal samples (Additional file 10: Dataset 5).

### Gene set enrichment analysis

The Broad Institute’s gene set enrichment analysis tool was used for this analysis. A gene set enrichment analysis was performed using the MSigDB database C6: Oncogenic Signatures. A key pathway, the ATF2_UP.V1_UP family, which contained 33 of our ranked genes, was found to have an enrichment score of 0.33, with a nominal *p* value of < 0.01, a false discovery rate of 5.7%, and an FWER *p* value of 0.052.

### Identification of CpG sites within gene promoters and bodies

Using the Table Browser function of the UCSC genome browser website, a database of 57,111 well-annotated coding and non-coding human genes was created from the RefSeq gene track and matched to genome build Hg19. The start and end positions of each gene were compared with the position of each differentially methylated CpG site using MATLAB scripts, allowing for the identification of CpG sites located within gene bodies, or in the promoter region of a gene (defined as the region 2 kb upstream of the transcription start site (TSS) of each gene) (Additional file 11).

### Test for non-randomness in clustering of DACOR1-affected genes

Arrays were created in MATLAB to record the relative position of differentially methylated CpG sites in relation to all CpG sites indexed by the RRBS assay. Following this, a MATLAB script parsed through each array to identify the number of differentially methylated CpG sites that were adjacent to each other when indexed by the RRBS assay. The MATLAB script then proceeded to generate a pseudorandom distribution of differentially methylated CpG sites within an array of size corresponding to the number of CpG sites indexed by the RRBS assay for that gene, and then counted the number of adjacent differentially methylated CpG sites as before, i.e., if in gene A, 100 CpG sites were differentially methylated within a gene body region containing 5000 CpG sites indexed by RRBS assay, a pseudorandom distribution of these 100 sites was generated, followed by a count of adjacent sites. This count would then be compared to the original count of adjacent sites (i.e., observed in data). This pseudorandom generation of sites followed by a count of adjacent differentially methylated sites was repeated 100,000 times for each gene or region of interest. *P* values were determined based on the number of trials where the count of adjacent sites from the pseudorandom distribution of differentially methylated sites exceeded or was equal to the count of adjacent sites from the observed data.

## Additional files

Additional file 1: **Figure S1.** DACOR1-mediated DNA methylation affects CpGs in Gene Bodies. A) Ten genes with the highest proportions of differential methylation post DACOR1 induction are plotted, with the positions and count of differentially methylated CpG per 1/100th of Gene Body length (bp set) visualized. B) For each gene, we mapped the position and relative count of all CpG sites within gene body regions. (TIF 90 kb)

Additional file 2: **Figure S2.** Network map linking key genes within the ATF2 family. A) Links between ATF2 (yellow), ATF2 family genes identified from our GSEA analysis (green), and related intermediaries (orange) are shown in a network map leading to developmental cell activity or angiogenesis/metastasis. Both inhibitory (purple lines) and activating (black lines) interactions were identified through peer-reviewed literature. (TIF 160 kb)

Additional file 3: Dataset 1: RRBS methylation data from colon tumors vs. normal colon samples. Dataset contains methylated CpG sites for 83 colon tumors and 40 normal colon samples. Additionally, gene names with corresponding counts of differential methylation are provided for gene bodies and promoter regions. (XLSX 797 kb)

Additional file 4: Dataset 7: RRBS assay of 83 Colon Tumors and 40 Normal Colon Samples. Complete information on CpG sites analyzed from RRBS assay of Colon Tumors and Normal Colon samples, filtered to sites with at least a 30% change in methylation with all referenced samples having at least 10 reads per site. (XLSX 121505 kb)

Additional file 5: Dataset 2: Colon Tumor vs. Normal Colon from TCGA (COAD) – processed methylation and overlap with expression data. Dataset contains distribution of methylated CpG sites for seven matched colon tumor and normal colon samples from HM450 assay raw data. Additionally, gene names with corresponding counts of differentially methylated CpG sites are provided for gene bodies and promoter regions. A comparison of differentially expressed genes from TCGA samples is then intersected with genes that show differential methylation. (XLSX 659 kb)

Additional file 6: Dataset 8: Differentially Methylated CpG sites from HM450 assays of TCGA COAD. Data on individual CpG sites identified as differentially methylated between matched Colon Tumor and Normal Colon samples from TCGA COAD. (XLSX 18415 kb)

Additional file 7: Dataset 5: Gene expression in colon tumors vs. matched normal colon samples (TCGA RNA-seq). Calculated FPKM value for each gene in each sample is provided. Analysis of differentially expressed and statistically significant genes is provided. (XLSX 35769 kb)

Additional file 8: Dataset 3: DACOR1-mediated changes in DNA methylation from RRBS data. Dataset contains distribution of methylated CpG sites obtained from RRBS assay of V852 cells that were transduced with a control LV or a DACOR1-expressing LV vector. Differentially methylated CpG sites are also presented with gene names and corresponding counts of differentially methylated CpG sites for both gene bodies and gene promoter regions. (XLSX 950 kb)

Additional file 9: Dataset 6: RRBS assay of V852 Cells with DACOR 1 Re-expression vs. control cells. Complete information on differentially methylated CpG sites from RRBS assay output of read values and read depth. (XLSX 2964 kb)

Additional file 10: Dataset 4: DACOR1-mediated changes in Gene Body methylation. Dataset contains locations of all differentially methylated CpG sites for top ten genes of interest, as well as identification if a specific site is located in an intron or an exon. (XLSX 34 kb)

Additional file 11: Dataset 10: Localization of modified CpGs. Modified CpGs according to their localization in CpG islands, CpG islands shores or shelves. (XLSX 774 kb)

Additional file 12: Dataset 9: RRBS Cohort demographic and Phenotypic data. Demographic and phenotypic information of the cohort analyzed in Fig. 1. (XLSX 14 kb)

## Abbreviations

bZIP: Basic leucine zipper
COAD: Colon adenocarcinoma
CpG: Cytosine-phosphate-guanine site
CREB: Cyclic AMP response element-binding
DNMT1: DNA methyltransferase 1
FPKM: Fragments per kilobase of transcript per million mapped reads
gDNA: Genomic DNA
lncRNA: Long non-coding RNA
RNA-seq: RNA sequencing
RRBS: Reduced representation bisulfide sequencing
TCGA: The Cancer Genome Atlas
TSS: Transcriptional start site
