# Supplementary figures and images for: The DNMT1-associated lincRNA DACOR1 reprograms genome-wide DNA methylation in colon cancer
Source: Clin Epigenetics. 2018 Oct 22;10:127. doi: 10.1186/s13148-018-0555-3 (PMC6196572; doi:10.1186/s13148-018-0555-3)

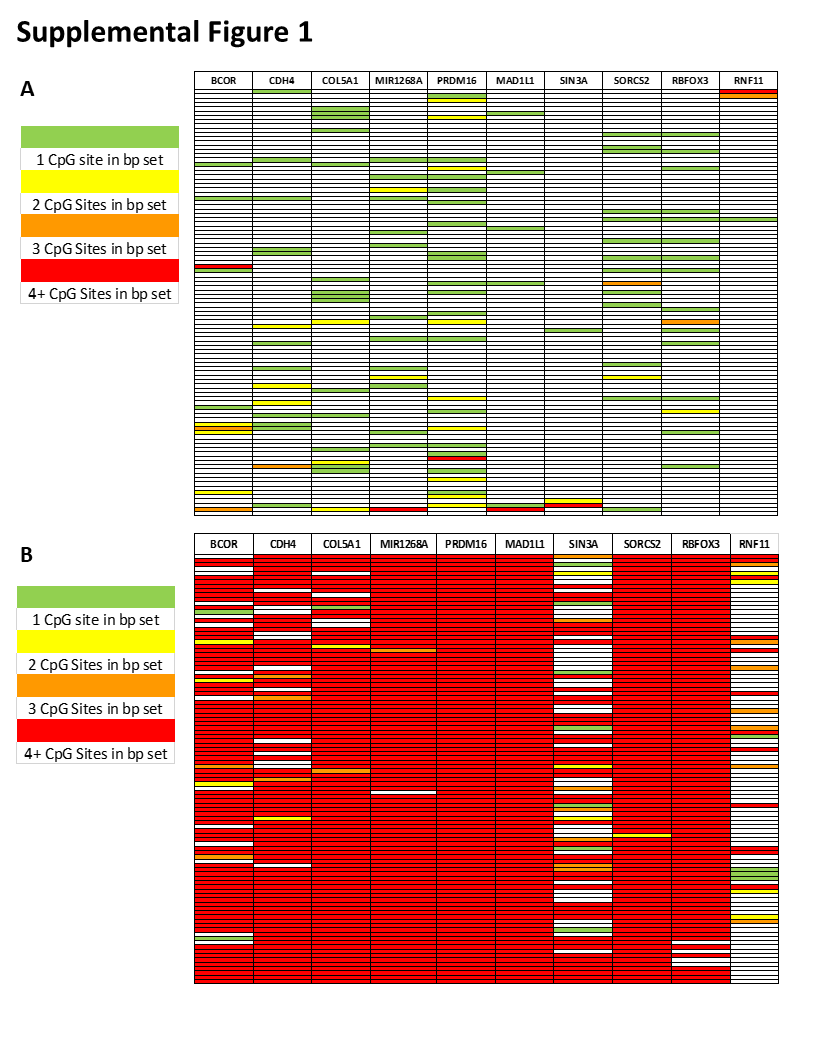

Supplement: Supplementary file 1 — Figure S1. DACOR1-mediated DNA methylation affects CpGs in Gene Bodies. A) Ten genes with the highest proportions of differential methylation post DACOR1 induction are plotted, with the positions and count of differentially methylated CpG per 1/100th of Gene Body length (bp set) visualized. B) For each gene, we mapped the position and relative count of all CpG sites within gene body regions. (TIF 90 kb) [file 13148_2018_555_MOESM1_ESM.tif]
